# 

**Published:** 1911-01

**Authors:** 


### CONTENTS.

ORIGINAL COMMUNICATIONS—
Artificial Pneumothorax in the Treatment of Pulmonary Tuberculosis. Mary E. Lapham, M. D., of the Highlands Camp Sanatorium, Highlands, N. C. .......... 493
Digestive Symptoms of Pellagra. By Joseph N. Le Conte, Atlanta, Ga. . 502
The Treatment of Anemia by Hydrotherapy—with Special Reference to the Anemia of Hook-Worm Disease. By William Lee Secor, Ph.D., M. D., Kerville, Texas, and Chicago .......... 507
Obstetric and Gynecologic Perineal Repair. By R. R. Kime, M. D., Atlatna, Ga. .......... 510
Inaugural Address. By Edgar G. Ballenger, M. D., Atlanta, Ga. .... 516
Self-Medication. By T. B. Rice, Ph.G., Greensboro, N. C. .......... 519
The Pathogenesis of Tabes Dorsalis. By Dr. T. A. Williams, Washington, D. C. .......... 523
EDITORIALS .......... 533
NEWS AND NOTES .......... 541
BOOK REVIEWS .......... 547

